# 2-Carba cyclic phosphatidic acid inhibits lipopolysaccharide-induced prostaglandin E2 production in a human macrophage cell line

**DOI:** 10.1016/j.bbrep.2019.100668

**Published:** 2019-07-19

**Authors:** Yuki Shibaike, Mari Gotoh, Chinatsu Ogawa, Shingo Nakajima, Keisuke Yoshikawa, Tetsuyuki Kobayashi, Kimiko Murakami-Murofushi

**Affiliations:** a Endowed Research Division of Beauty and Science, Ochanomizu University, 2-1-1 Ohtsuka, Bunkyo-ku, Tokyo, 112-8610, Japan; b Research Organization for the Promotion of Global Women's Leadership, Ochanomizu University, 2-1-1 Ohtsuka, Bunkyo-ku, Tokyo, 112-8610, Japan; c Institute for Human Life Innovation, Ochanomizu University, 2-1-1 Ohtsuka, Bunkyo-ku, Tokyo, 112-8610, Japan; d Graduate School of Humanities and Sciences, Ochanomizu University, 2-1-1 Ohtsuka, Bunkyo-ku, Tokyo, 112-8610, Japan; e Department of Mental Disorder Research, National Institute of Neuroscience, National Center of Neurology and Psychiatry (NCNP), 4-1-1, Ogawa-Higashi, Kodaira, Tokyo, Japan; f Department of Pharmacology, Faculty of Medicine, Saitama Medical University, 38 Moro-hongo, Moroyama-machi, Iruma-gun, Saitama, 350-0495, Japan

**Keywords:** 2ccPA, Prostaglandin E2, Anti-inflammatory, THP-1 monocytes

## Abstract

Cyclic phosphatidic acid (cPA) is a naturally occurring phospholipid mediator that contains a unique cyclic phosphate ring at the *sn*-2 and *sn*-3 positions of its glycerol backbone. Using mouse models for multiple sclerosis (cuprizone-induced demyelination and experimental autoimmune encephalomyelitis) and traumatic brain injury, we revealed that cPA and its metabolically stabilized cPA derivative, 2-carba-cPA (2ccPA), have potential to protect against neuroinflammation. In this study, we investigated whether 2ccPA has anti-inflammatory effect on peripheral immune function or not using inflammation-induced macrophages-like cell line, THP-1 monocytes differentiated by phorbol 12-myristate 13-acetate (PMA). Lipopolysaccharide (LPS)-stimulated THP-1 cells were found to have higher expression of the mRNAs of several inflammation-related cytokines and of the enzyme cyclooxygenase-2 (Cox-2); however, when THP-1 cells were stimulated by LPS in the presence of 2ccPA, the increase in the expression of pro-inflammatory cytokine and Cox-2 mRNA was attenuated. 2ccPA treatment also decreased the amount of prostaglandin E2 (PGE2) produced by LPS-stimulated THP-1 cells and decreased expression of the mRNA of prostaglandin E receptor 2 (EP2, *PTGER2*), a PGE2 receptor that mediates inflammation. These results indicate that 2ccPA has anti-inflammatory properties.

## Highlights

• 2-Carba cyclic phosphatidic acid inhibits prostaglandin E2 production.
• 2-Carba cyclic phosphatidic acid has anti-inflammatory effect.
• 2-Carba cyclic phosphatidic acid has effect on peripheral immune function.

## 1 Introduction

Macrophages play important roles in the host defense system *via* the phagocytosis and release of inflammation-associated enzyme (cyclooxygenase-2, Cox-2) and various cytokines, such as pro- (interleukin [IL]-1β, IL-6, tumor necrosis factor [TNF]-α) and anti-inflammatory cytokines (transforming growth factor [TGF]-β) [1,2]. Cox-2 is an enzyme that synthesizes prostaglandin (PG) E2, a lipid mediator, which is responsible for inflammation and pain [3]; this enzyme is therefore one of the targets of non-steroidal anti-inflammatory drugs. Moreover, the release of pro-inflammatory cytokines is also responsible for systemic inflammation. We therefore surmise that attenuating the expression of Cox-2 and pro-inflammatory cytokines in activated macrophages could relieve inflammation.

Cyclic phosphatidic acid (cPA) is a lysophospholipid structurally similar to lysophosphatidic acid (LPA), first isolated from the myxamoebae of a true slime mold, *Physarum polycephalum*, in 1992 [4]. cPA was later found in mammalian tissues and normal and diseased serum [[5], [6], [7], [8], [9], [10]]. We previously reported that cPA attenuates neuropathic pain and neuroinflammation [11,12]. These results suggested that cPA could be a therapeutic compound for these diseases. To develop this idea, we synthesized several cPA derivative compounds. 2ccPA is one of the stabilized derivative compounds of cPA in which the phosphate oxygen has been replaced with a methylene group at the *sn*-2 position [[13], [14], [15]]. Our previous study has demonstrated that 2ccPA administration reduced demyelination in a mouse model of multiple sclerosis *via* suppression of microglial activation and the NLRP3 inflammasome [16]. Moreover, 2ccPA reduced the damage associated with traumatic brain injury (TBI) induced using stab wound surgery with vertical needle injury and suppressed the expression of pro-inflammatory cytokines (IL-1β, IL-6, and TNF-α) [17]. Although these experiments have demonstrated the anti-inflammatory effects of 2ccPA on the central nervous system (CNS), it is still unclear whether 2ccPA exerts anti-inflammatory effects in the peripheral immune system.

In this study, to gain insight into the anti-inflammatory function of 2ccPA upon exposure to infectious stimuli, we investigated the effect of 2ccPA on cytokines and Cox-2 mRNA expression using a lipopolysaccharide (LPS)-stimulated human macrophage cell line. In our experiment, we found that 2ccPA attenuated the increase in the expression of Cox-2 mRNA. Therefore, we examined the effect of 2ccPA on arachidonic cascade and found that 2ccPA has regulatory effect on the production of members of the arachidonic cascade.

## 2 Materials and methods

### 2.1 Pharmacological agents

1-oleoyl-2-O-carba-cPA (2ccPA 18:1) was chemically synthesized as previously described []. Bovine serum albumin (BSA; fraction V, fatty acid free) and lipopolysaccharide (LPS; from *Escherichia coli* O111:B4) were purchased from Sigma-Aldrich (St. Louis, MO). 2ccPA was dissolved in phosphate buffered-saline (PBS) containing 0.1% (w/v) BSA at final concentrations of 1 mM.

### 2.2 Cell culture and treatment

THP-1 cells, purchased from JCRB Cell Bank (Osaka, Japan), were cultured with RPMI1640 containing 10% heat-inactivated fetal bovine serum (FBS, Biowest, FL) at 37 °C (95% humidity, 5% CO_2_). Differentiation into macrophages was induced by the addition of 100 nM of phorbol 12-myristate 13-acetate (PMA, Wako, Tokyo, Japan) to the cells, and then cells were incubated for 48 h. The culture medium was replaced with fresh serum-free RPMI1640 to achieve serum starvation. After 16 h, 1 μg/mL of LPS was added to the differentiated THP-1 cells. For experiments to assess the effect of 2ccPA on the cells, various concentrations of 2ccPA or vehicle (0.1% BSA/PBS) was added together with LPS.

### 2.3 Western blot analysis

THP-1 cells were collected using 0.125 M Tris-HCl (pH 6.8) containing 4% sodium dodecyl sulfate, 20% glycerol, 10% 2-mercaptoethanol and 0.01% bromophenol blue. Proteins were separated using a 10% acrylamide gel in a SDS-PAGE, and then the separated proteins were transferred to an Immobilon-P Transfer Membrane (Millipore). After blocking with 5% skim milk for 1 h, the membrane was washed several times with Tris buffered saline containing 0.1% Tween 20 (TBST). Primary antibody for anti-CD11b antibodies (1:1000 dilution, Cell Signaling Technology, Inc., MA) or with anti-GAPDH antibodies (1:1000 dilution, Cell Signaling Technology, Inc.) diluted with Can Get Signal immunoreaction enhancer solutions (TOYOBO Co., LTD., Osaka, Japan) were incubated overnight. Next, following three rounds of washing with TBST, it was treated with horseradish peroxidase-conjugated anti-rabbit IgG (1:2500 dilution; Cell Signaling Technology, Inc.) for 2 h. Immunodetection was performed using an enhanced chemiluminescence system (Bio-Rad Laboratories, Inc., CA) and ImageQuant LAS 4000 (GE healthcare UK Ltd., Buckinghamshire, UK).

### 2.4 Measurement of PGE2 produced by differentiated THP-1 cells

To measure the concentration of secreted PGE2, LPS (1 μg/mL) was applied along with 2ccPA or vehicle (0.1% BSA/PBS) after serum starvation at final concentrations of 1, 3, and 10 μM. After 6 h, the culture media were collected and PGE2 concentration was measured using ELISA (R&D Systems, Inc. MN) according to the manufacturer's protocol.

### 2.5 Quantitative real-time reverse transcription-polymerase chain reaction (RT-PCR)

Total RNA was extracted from cultured cells using TRI reagent^®^ (Molecular Research Center, Inc., OH) according to the manufacturer's instructions. cDNA was synthesized using a PrimeScript RT reagent kit (Takara Bio, Inc.). To quantify the mRNA levels of the genes of interest and LPA receptors, real-time RT-PCR was performed using the THUNDERBIRD SYBR qPCR Mix (Toyobo, Osaka, Japan) and the lightCycler 96 system (Roche, Tokyo, Japan). Gene-specific primer sets for LPA receptors have been described in a previous report [18]. Other primer sets are described in Table 1. The data were calculated based on the Cq values and the expression of each gene was normalized to that of GAPDH.

**Table 1:** 

Primer sequences used for real-time RT-PCR		
Gene	Primer Sequence	
Cd11b *(ITGAM)*	Forward	5′-AGAACAACATGCCCAGAAC-3′
	Reverse	5′-GCGGTCCCATATGACAGTCT-3′
IL-1α *(IL1A)* [23]	Forward	5′- CGCTCAAGATGAAGGCAAAG -3′
	Reverse	5′- GATGCCTGGTCACACTCAGA -3′
IL-1β (*IL1B*) [24]	Forward	5′-GCACGATGCACCTGTACGAT -3′
	Reverse	5′- AGACATCACCAAGCTTTTTTGCT-3′
IL-6 (*IL6*) [24]	Forward	5′- GTGAAAGCAGCAAAGAGGCACT-3′
	Reverse	5′-ACCAGGCAAGTCTCCTCATTGA -3′
IL-8 *(CXCL8)* [25]	Forward	5′- AAGGAACCATCTCACTGTGTGTAAAC-3′
	Reverse	5′- ATCAGGAAGGCTGCCAAGAG -3′
TNF-α (*TNF*) [24]	Forward	5′-TGAGTCCTGCTCCTTCCA-3′
	Reverse	5′-GCTTCGGTGTAGCCCATT-3′
Cox-2 *(PTGS2)* [26]	Forward	5′- CAGCACTTCACGCATCAGTT-3′
	Reverse	5′-CGCAGTTTACGCTGTCTAGC-3′
TGF-β *(TGFB1)* [24]	Forward	5′- CAAGGGCTACCATGCCAACT-3′
	Reverse	5′- AGGGCCAGGACCTTGCTG-3′
IL1RA *(IL1RN)* [27]	Forward	5′- -GGAATCCATGGAGGGAAGAT-3′
	Reverse	5′- -CCTTCGTCAGGCATATTGGT-3′
IL-10 *(IL10)* [24]	Forward	5′- ATGCTGCCTGCTCTTACTGACTG-3′
	Reverse	5′-CCCAAGTAACCCTTAAAGTCCTGC -3′
EP2 (*PTGER2*) [28]	Forward	5′-CTGCTGCTGCTTCTCATTGT-3′
	Reverse	5′-ATGCGGATGAGGTTGAGAAT-3′
EP4 *(PTGER4)* [28]	Forward	5′-CGACCTTCTACACGCTGGTATG-3′
	Reverse	5′-CCGGGCTCACCAACAAAGT-3′

### 2.6 Statistical analyses

All values are reported as mean ± standard error. Statistical analysis was performed using Prism 6 (GraphPad). Significant difference was assessed by the Student's *t*-test for pair comparison and Bonferroni's correction for multiple comparisons. All experiments were independently repeated at least 4 times and representative data are shown.

## 3 Results and discussion

### 3.1 Altered expression levels of LPA receptors (LPARs) during THP-1 cell differentiation

**Fig. 1:**
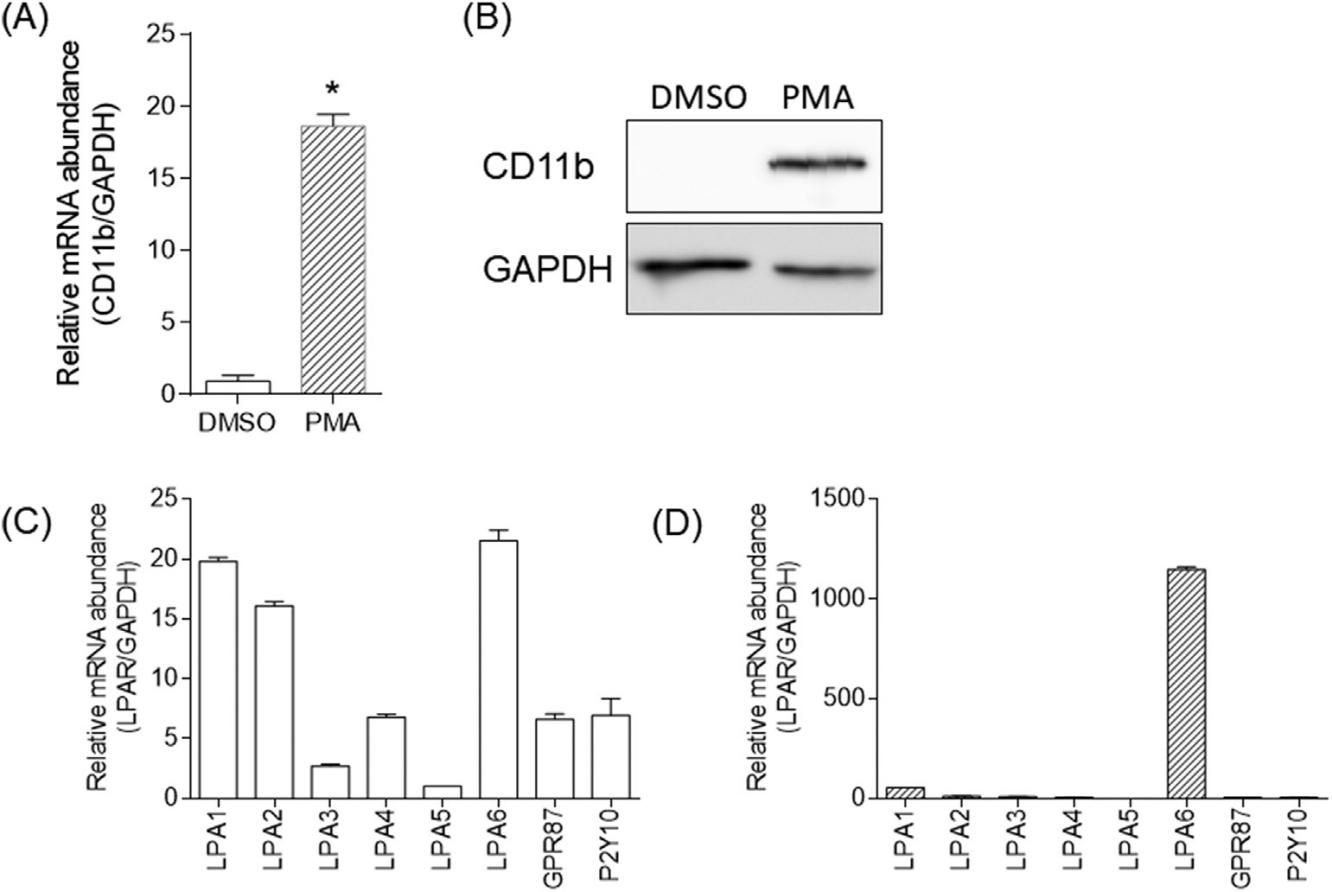
CD11 and LPAR expression in PMA-differentiated THP-1 cells. (A) The expression level of CD11b mRNA was determined using real-time RT-PCR. Data are presented as the mean ± SEM. Statistical analysis was performed using the Student's *t* test. (*p < 0.0001, vs. DMSO vehicle control). (B) The protein levels of CD11b and GAPDH were determined using Western blot analysis. Real-time RT-PCR analyses of LPA1, LPA2, LPA3, LPA4, LPA5, LPA6, GPR87, and P2Y10 mRNA expression levels in (C) THP-1 cells and (D) differentiated THP-1 cells. The mRNA expression level of each gene was normalized relative to that of GAPDH.

PMA (100 nM) treatment for 48 h morphologically changed THP-1 cells to adhere to the plastic culture dishes. We collected the adherent cells and confirmed the mRNA and protein expression of CD11b, a pan-macrophage marker. Because CD11b expression was increased in PMA-treated THP-1 cells (Fig. 1A and B), we used these adherent cells as being representative of human macrophage for subsequent experiments.

To investigate the effect of 2ccPA on PMA-treated THP-1 cells, we first investigated the mRNA expression of LPA receptors, which are considered candidate targets of 2ccPA, in THP-1 and PMA-treated THP-1 cells (Fig. 1C and D). In THP-1 cells, LPA1, LPA2, and LPA6 mRNA expression were higher than those of other LPA receptors. In PMA-treated THP-1 cells, the mRNA expression of LPA6 increased apparently. It is known that LPA6 couples to G12/13, and induces actin stress fiber formation *via* G12/13/RhoA/ROCK activation [19]. These results suggest that when circulating monocytes become adherent macrophages upon treatment with PMA, increased expression of LPA6 might be required to control the formation of actin stress fibers.

### 3.2 Expression of genes for inflammation-related cytokines and enzyme

**Fig. 2:**
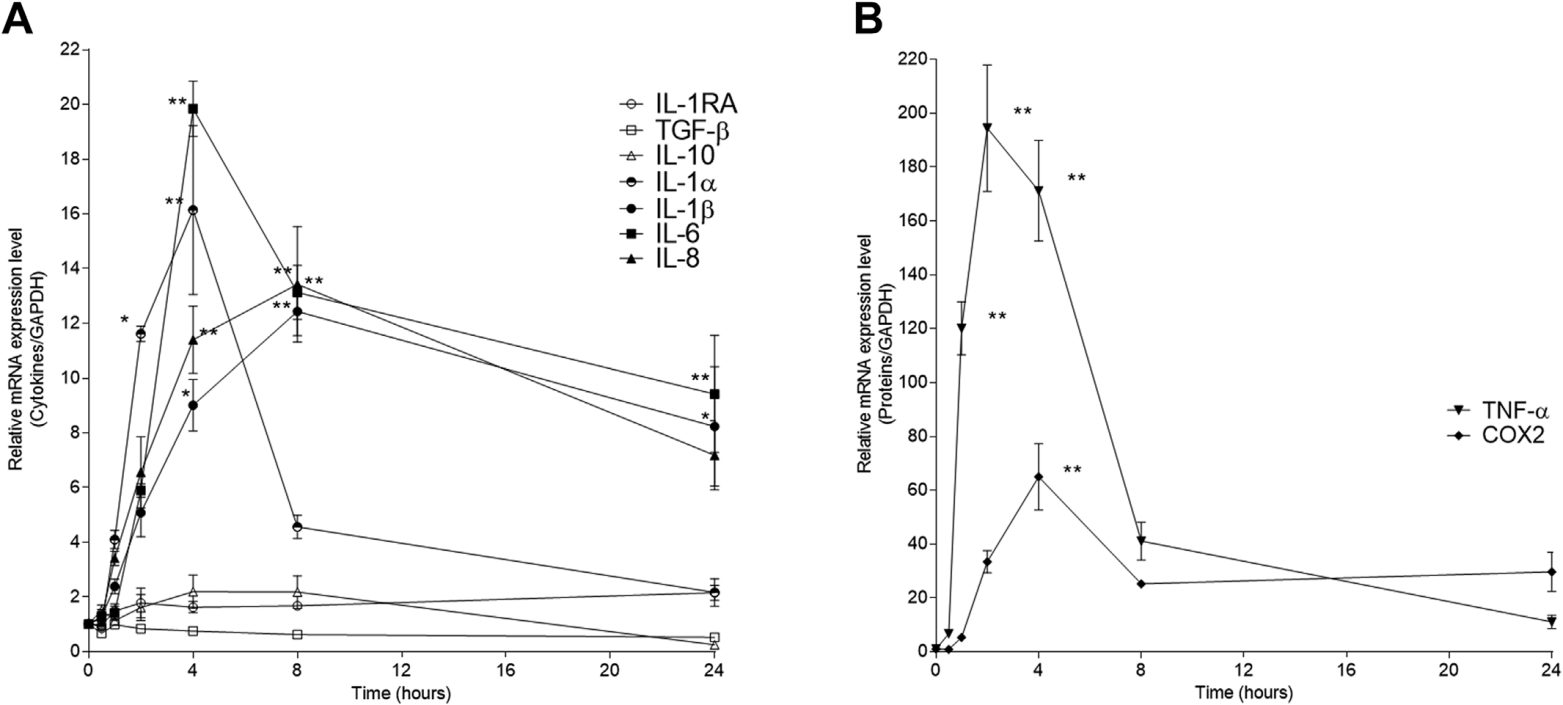
Variation in cytokine and Cox-2 mRNA expression after LPS stimulation in differentiated THP-1 cells. To measure mRNA expression, differentiated THP-1 cells were cultured with LPS (1 μg/mL) for the time indicated. (A) IL-1RA, TGF-β, IL-10, IL-1α, IL-1β, IL-6, IL-8, and (B) TNF-α and Cox-2 mRNA levels were determined using quantitative real-time PCR. Data represent the mean ± SEM of triplicate independent experiments. One-way ANOVA followed by Bonferroni's post-hoc comparisons tests were performed. (*p < 0.001, **p < 0.0001 vs. 0 time).

To induce an inflammatory response, the PMA-treated THP-1 cells were stimulated with LPS. In our study, the mRNA expression levels of anti-inflammatory cytokines (IL-1RA IL-10, TGF-β) did not increase following LPS exposure; however, those of all pro-inflammatory cytokines (IL-1α, IL-1β, IL-6, IL-8, and TNF-α) increased (Fig. 2A). The fold changes in the mRNA expression of TNF-α and Cox-2, PEG2 synthesizing enzyme, were remarkable (Fig. 2B). Maximal expression of most pro-inflammatory cytokines was observed after around 4 h of stimulation, except TNF-α, the maximal expression of which was achieved after 2 h of stimulation.

### 3.3 Effect of 2ccPA on inflammatory functions of PMA-treated THP-1 cells

**Fig. 3:**
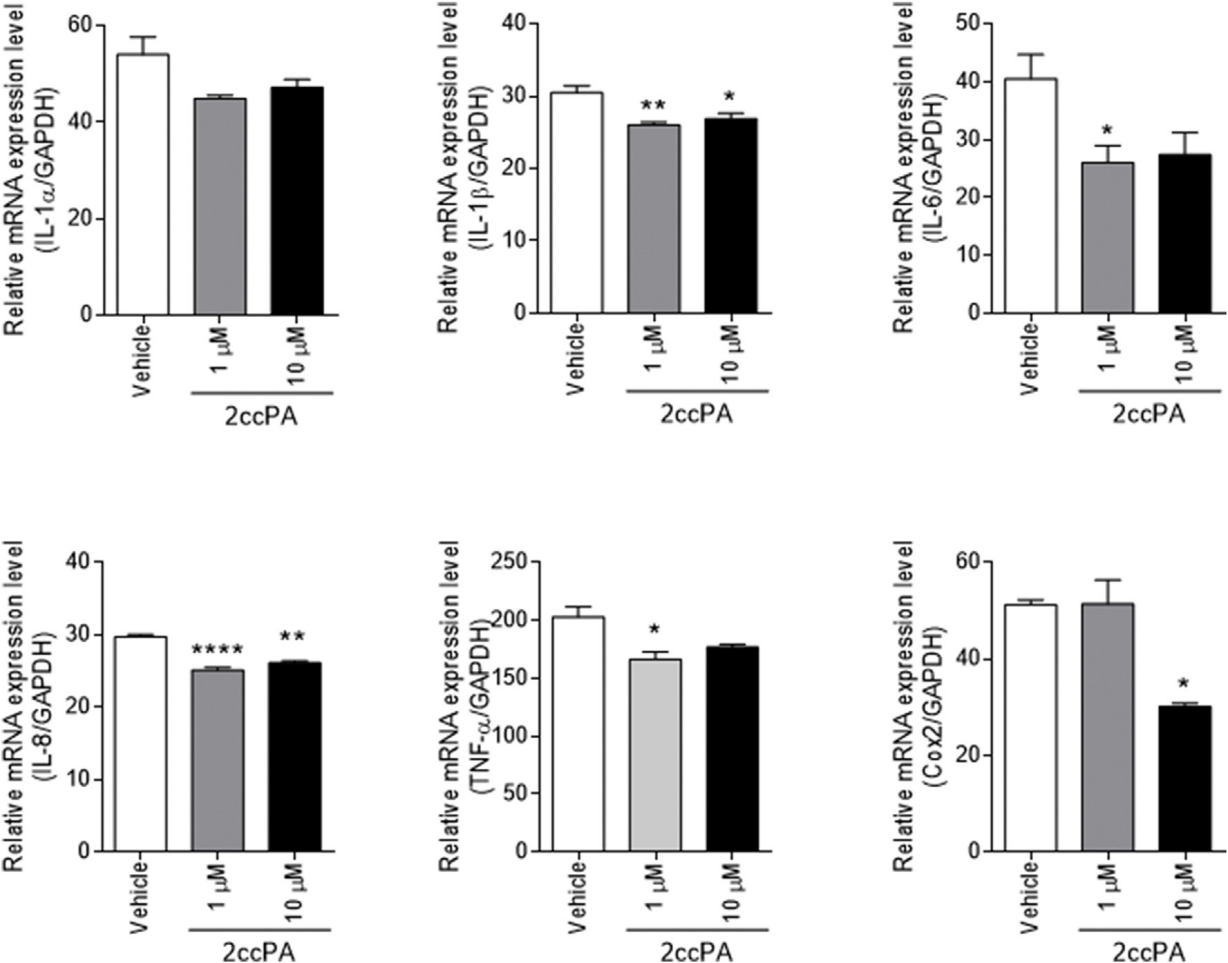
Effect of 2ccPA on LPS-induced cytokines and Cox-2 mRNA expression and PGE2 secretion in differentiated THP-1 cells. Differentiated THP-1 cells were incubated with LPS (1 μg/mL) along with or without 2ccPA (1, 10 μM). Cells were collected after 4 h of incubation and mRNA expressions were analyzed by real-time PCR. The data were calculated based on the Cq values, and the expression of each gene was normalized to the levels of GAPDH. The data were calculated as the relative induction compared to that of controls (without LPS treatment). Data are presented as the mean ± SEM, n = 4. Statistical analysis was performed using a Bonferroni's test. (*p < 0.05; **p < 0.01, ***p < 0.001, ****p < 0.0001 vs. Vehicle).

To investigate the effect of 2ccPA on LPS-induced pro-inflammatory cytokine expression in macrophages, PMA-treated THP-1 cells were simultaneously exposed to 2ccPA and LPS. The addition of 2ccPA attenuated the increased expression of IL-1B, IL-6, IL-8, TNF-α, and Cox-2 mRNA caused by the exposure to LPS, particularly for IL-6 and Cox-2 (Fig. 3). 2ccPA had no effect on the mRNA expression of anti-inflammatory cytokines with or without LPS stimulation (data not shown).

**Fig. 4:**
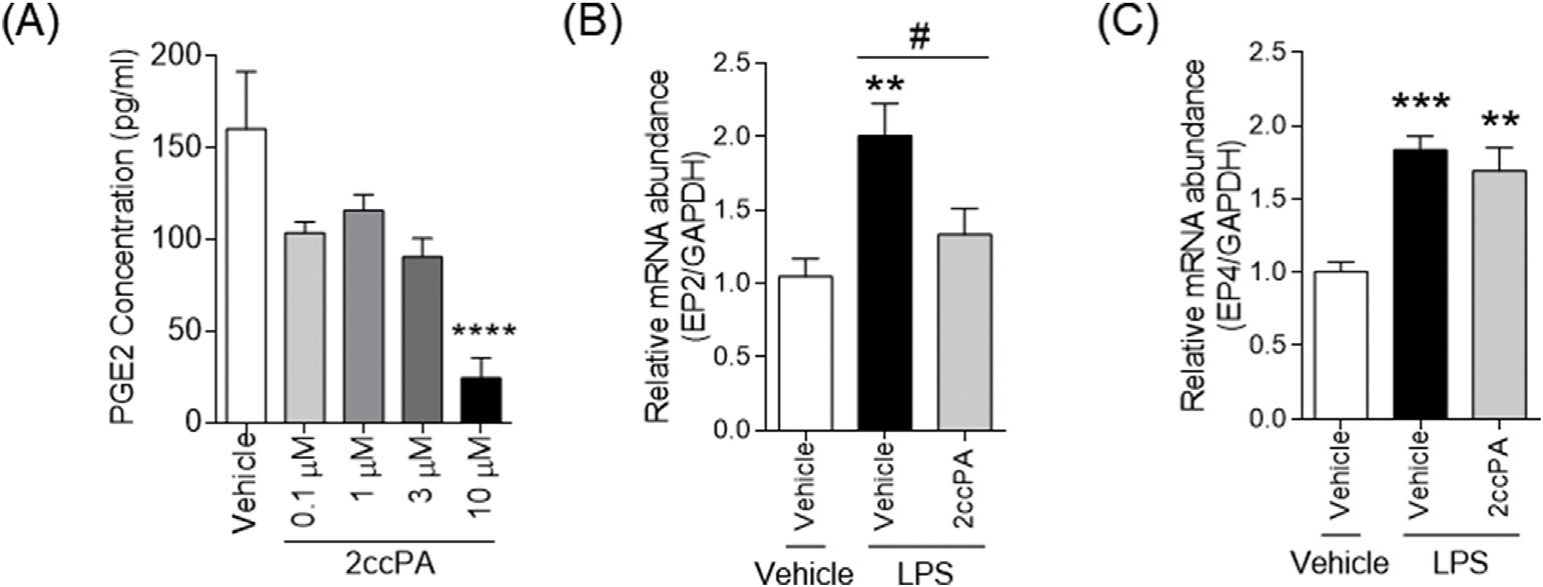
Effect of 2ccPA on LPS-induced PGE2 secretion and PGE2 receptor mRNA expression in differentiated THP-1 cells. (A) Effect of 2ccPA on PGE2 secretion in differentiated THP-1 treated with LPS. Differentiated THP-1 cells were incubated with LPS (1 μg/mL) along with 2ccPA (0.1, 1, 3, or 10 μM) for 6 h. The concentrations of PGE2 in culture media were determined using ELISA. One-way ANOVA followed by Bonferroni's post-hoc comparisons tests were performed. (****p < 0.0001 vs. Vehicle). (B&C) Differentiated THP-1 cells were incubated with LPS (1 μg/mL) in the presence of 2ccPA (10 μM) for 4 h. The cells were collected and the Ep2 (B) and Ep4 (C) mRNA expressions were analyzed by real-time PCR. (*p < 0.05; **p < 0.01 vs. Vehicle without LPA, ^#^*P* < 0.05; vs. Vehicle with LPA).

Cox-2 is an enzyme involved in the synthesis of prostaglandin (PG) E2, a lipid mediator, which is responsible for inflammation and pain [3]. Therefore, we focused on the effect of 2ccPA on the attenuation of Cox-2 mRNA expression. As expected, 10 μM of 2ccPA significantly decreased the release of PGE2 from macrophages (Fig. 4A).

We also investigated the expression of PGE2 receptors. PGE2 acts *via* four specific G protein-coupled receptors (EP1-4). Although the expression level of EP1 and EP3 receptor mRNA expression was very low or undetectable in PMA-treated THP-1 cells (data not shown), the expression of EP2 and EP4 mRNA increased following LPS stimulation (Fig. 4B and C). EP2 signaling is reported to be associated with both pro- and anti-inflammatory effects upon LPS stimulation [20], while EP4 receptors are known to play a role in the anti-inflammatory response [21]. Interestingly, the increase in EP2 mRNA expression caused by LPS was abolished by co-treatment with 2ccPA, whereas 2ccPA did not affect the LPS-stimulated increase in EP4 mRNA expression (Fig. 4B and C). These results indicate that 2ccPA may modulates inflammation by downregulating Cox-2 mRNA expression and thereby PGE2 production. Remarkably, we had previously demonstrated that 2ccPA decreases cuprizone-induced demyelination and motor dysfunction [16]. The increase in the expression levels of EP2 receptor has been suggested to parallel the increase in Cox-2 mRNA expression in a cuprizone-induced demyelination mouse model [22]. Although it is still unclear whether the regulation of the peripheral immune system by 2ccPA contributes to the suppression of neuronal inflammation, 2ccPA might decrease the expression level of Cox-2 and EP2 mRNA, and the resultant decrease in the expression of PGE2 could partially contribute to the attenuation of neuroinflammation.

In summary, LPS-induced inflammation in PMA-treated THP-1 cells was attenuated by co-treatment with 2ccPA. Further, 2ccPA decreased the expression of mRNA of inflammatory enzyme Cox-2, and thereby the production of PGE2. These results revealed that 2ccPA has regulatory effects on the arachidonic acid cascade, which suggests the potential therapeutic effects of 2ccPA on inflammatory disease through its anti-inflammatory functions.

## COI

The authors declare no conflicts of interest associated with this manuscript.
